# A Commentary on the “Global Consensus on Optimal Exercise Recommendations for Enhancing Healthy Longevity in Older Adults (ICFSR)”: Exercise and older adults—A perfect match

**DOI:** 10.1016/j.jnha.2024.100414

**Published:** 2025-01-01

**Authors:** Michael Pratt

**Affiliations:** Herbert Wertheim School of Public Health & Human Longevity Science, University of California San Diego, La Jolla, CA, United States

Congratulations to the authors of the Global Consensus on Optimal Exercise Recommendations for Enhancing Healthy Longevity in Older Adults (ICFSR) [1]. This is an incredibly important public health and clinical issue. Nobody stands to benefit more from regular exercise or physical activity than older adults. While excellent national and global recommendations for physical activity exist and do include and even emphasize physical activity for older adults there is space for a more focused document such as this one [2,3]. Why do we need another review and set of recommendations for exercise in older adults? As the evidence base rapidly evolves particularly in areas such as physical activity and metabolic changes with aging, technology as a tool for promoting physical activity, and cognition and physical activity among older adults our consensus statements should reflect these advances in understanding the complex interplay of aging, exercise and health. It is equally important to regularly update or reinforce clinical and public health recommendations in order to counter the constant flow of disinformation that exists in our modern media environment. Absent regular evidence-based updates from legitimate sources the discourse on any health topic can go awry.

It is impossible in a brief commentary to touch on the full breadth of issues covered in this extensive report. However, I will comment on a few aspects of physical activity among older adults that I believe are especially important. In no other age group is the full breadth of exercise and physical activity (aerobic, resistance, flexibility, balance) more impactful. Preventing and treating non-communicable diseases, maintaining functional capacity, avoiding falls, and preserving cognition are critical for older adults. Tailoring the right mix of aerobic, strength, flexibility, and balance “training” for older adults is a substantial challenge that few health care systems currently handle well. While our understanding of the science of aging and physical activity continues to advance, we seem to be stuck in about the same place when it comes to translating science to improve the health and quality of life of older adults across the age, economic, and cultural spectra. Yes, promoting physical activity among older adults is more challenging due to underlying disease, higher risks, overuse of medications, and the much greater need to tailor interventions to go beyond aerobic activity. As wonderful as walking programs are for the vast majority of people, they don’t fulfill all of the needs of older adults.

So, what can be done? First, we must double down on training and education for both health professionals and the public. Effective promotion of physical activity among older adults requires a multi-disciplinary approach and our education efforts must go beyond physicians. Titrating the role of physicians to optimize their impact and minimize their time commitment is essential. Of course, this means that other health and exercise professionals must be seamlessly connected within health care systems and the communities where exercise and physical activity actually take place. Every country is likely to have a slightly different approach to do this and sharing these examples through networks such as HEPA Europe can be helpful [4]. Exercise is Medicine is another example of a program that has been adapted in multiple countries to bridge clinical and community settings [5]. The public can play a very important role as well by creating demand for high quality integrated and tailored programs with wide access. Second, we should strategically address referral from clinical to community settings. This is crucial for physical activity promotion across all age groups but even more so for older adults who especially benefit from input from multiple specialists focused on the several fitness and health components that impact their health, wellness and functional capacity. Finally, as noted towards the end of this report we need a much greater emphasis on implementation science. Physical activity promotion for older adults is a classic demonstration of the need for dissemination and implementation research to guide practice. We have strong and compelling evidence for physical activity promotion for older adults coupled with suboptimal delivery in both public health and clinical spheres. Building on the ever-expanding evidence based nicely summarized in this report to increase the translation of that science into high quality practice in clinics and communities is a worthy and achievable goal.

## Declaration of interests

The authors declare that they have no known competing financial interests or personal relationships that could have appeared to influence the work reported in this paper.
